# Ionic Liquid-Based Silane for SiO_2_ Nanoparticles: A Versatile Coupling Agent for Dental Resins

**DOI:** 10.1021/acsami.4c04580

**Published:** 2024-06-24

**Authors:** Isadora Martini Garcia, Virgínia Serra de Souza, Abdulrahman A. Balhaddad, Lamia Mokeem, Mary Anne Sampaio de Melo, Jackson Damiani Scholten, Fabrício Mezzomo Collares

**Affiliations:** † Division of Cariology and Operative Dentistry, Department of Comprehensive Dentistry, University of Maryland School of Dentistry, Baltimore, Maryland 21201, United States; ‡ Laboratory of Molecular Catalysis, Institute of Chemistry, Federal University of Rio Grande do Sul, Bento Gonçalves Avenue, 9500, Agronomia, 91501-970 Porto Alegre, Rio Grande do Sul, Brazil; § Department of Restorative Dental Sciences, College of Dentistry, Imam Abdulrahman Bin Faisal University, P.O. Box 1982, 31441 Dammam, Saudi Arabia; ∥ Dental Biomedical Sciences Ph.D. Program, University of Maryland School of Dentistry, Baltimore, Maryland 21201, United States; ⊥ Laboratory of Molecular Catalysis, Institute of Chemistry, Federal University of Rio Grande do Sul, Bento Gonçalves Avenue, 9500, Agronomia, 91501-970 Porto Alegre, Rio Grande do Sul, Brazil; # Department of Dental Materials, School of Dentistry, Federal University of Rio Grande do Sul, Ramiro Barcelos Street, 2492, Rio Branco, 90035-003 Porto Alegre, Rio Grande do Sul, Brazil; ¶ Dental Materials Laboratory, School of Dentistry, Federal University of Rio Grande do Sul, 90035-003 Porto Alegre, Rio Grande do Sul, Brazil

**Keywords:** silanes, ionic liquids, composite dental resin, nanocomposites, polymerization, mechanical tests, microbial sensitivity tests

## Abstract

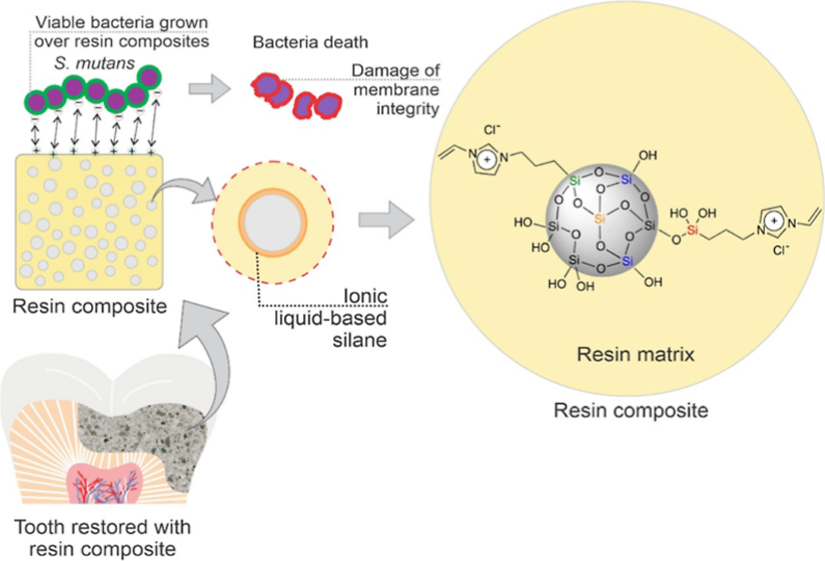

The current longevity of dental resins intraorally is limited by susceptibility to acidic attacks from bacterial metabolic byproducts and vulnerability to enzymatic or hydrolytic degradation. Here, we demonstrate synthesizing an ionic liquid-based antibiofilm silane effective against *Streptococcus mutans*, a major caries pathogen. Furthermore, we incorporate this silane into dental resins, creating antibiofilm- and degradation-resistant materials applicable across resin types. FTIR, UV–vis, and NMR spectroscopy confirmed the synthesis of the expected ionic liquid-based silane. The characterization of SiO_2_ after the silanization indicated the presence of the silane and how it interacted with the oxide. All groups achieved a degree of conversion similar to that found for commercial resin composites immediately and after two months of storage in water. The minimum of 2.5 wt % of silane led to lower softening in solvent than the control group (G_CTRL_) (*p* < 0.05). While the flexural strength indicated a lower value from 1 wt % of silane compared to G_CTRL_ (*p* < 0.05), the ultimate tensile strength did not indicate differences among groups (*p* > 0.05). There was no difference within groups between the immediate and long-term tests of flexural strength (*p* > 0.05) or ultimate tensile strength (*p* > 0.05). The addition of at least 5 wt % of silane reduced the viability of *S. mutans* compared to G_CTRL_ (*p* < 0.05). The fluorescence microscopy analysis suggested that the higher the silane concentration, the higher the amount of bacteria with membrane defects. There was no difference among groups in the cytotoxicity test (*p* > 0.05). Therefore, the developed dental resins displayed biocompatibility, proper degree of conversion, improved resistance against softening in solvent, and stability after 6 months of storage in water. This material could be further developed to produce polymeric antimicrobial layers for different surfaces, supporting various potential avenues in developing novel biomaterials with enhanced therapeutic characteristics using ionic liquid-based materials.

## 1 Introduction

Dental restoration failures and consequent restoration replacement cause high economic costs^1^ and significant losses of healthy dental tissues over time.^2^ In addition, the reintervention and the subsequent restoration increase the risk of pulp injury, weaken the tooth reminiscent, increase the size of the restoration, and raise the chances of restoration failure.^2^ Despite improving dental materials over the years, the longevity of restorations is still a concern, mainly due to the development of caries at the margin adjacent to the resin composites.^3,4^

Recurrent caries is a major factor in premature failures of composites after 5 years of clinical service.^5^ Patients at high risk of caries present an annual failure rate (AFR) of 4.6% in 10 years, while those at low risk show an AFR of 1.6% in the same period. In this scenario, while low-risk patients have about 60% restoration survival chances, those at high risk have a survival of only 35% in 20 years.^5^ To overcome this issue, restorative materials have been modified to present antibacterial activity. Quaternary ammonium compounds, antibacterial oxides, antibacterial peptides, and nanocarriers with antibacterial drugs (such as chlorhexidine or amoxicillin) are the most tested so far.^6,7^ While some of these materials exhibit promising results, the majority lack the chemical versatility required, for instance, to be modified over time to overcome antimicrobial resistance.

Ionic liquids have been shown to be promising antimicrobial agents for dental materials. These salts present a low melting point (below 100 °C) and can be found in a liquid state even under room temperature.^8^ They comprise a cationic core, usually an organic group with nitrogen (such as imidazole), linked to an alkyl chain. This positively charged structure chemically interacts with anionic species, such as chloride.^8^ The first ionic liquid at room temperature (ethylammonium nitrate) was synthesized in 1914.^9^ Initially, these materials were studied, considering only their physical and chemical properties. From the 1990s onward, significant advances in understanding the biological properties of ionic liquids boosted their application in the health sciences.^10^

Moreover, their versatility and potential for multiple functionalities depending on the cation and anion chosen increased their request.^8,10^ In dentistry, ionic liquids were initially investigated to protect the titanium surfaces of implants against bacterial colonization and corrosion.^11^ Later, they were used to coat silver nanoparticles to disinfect root canals against *Enterococcus faecalis*.^12^ Recently, we functionalized quantum dots and used them as nanofillers into dental adhesives^13,14^ to confer antibacterial activity for the polymer and nanoparticle size stability. However, despite advances, the synthesis of ionic liquids able to covalently bond with the resin is a gap that must be surpassed to make the nanoparticles chemically attached to the organic matrix.

In this study, we proposed the synthesis of a polymerizable ionic liquid to replace an essential reagent of all resin composites used in dental materials: silanes. Resin composites are the main materials used for dental restorations. These materials are comprised of inorganic fillers, monomers, initiators, pigments, and a coupling agent, which is usually an organic silicon combination with functional groups.^15^ The most used coupling agent is 3-methacryloxypropyltrimethoxysilane (MPTS),^16^ which is covalently bound on one side to the inorganic filler and on the other side to the resin matrix through its methacrylate group.^15^ They are essential reagents for many materials in dentistry, such as filled adhesives, cements, sealers, cements, sealants, and resin composites. Silanes are very important as they act as a bridge between the inorganic and organic phases of the composite, improving the transfer of stress when the composite is tensioned and reducing hydrolytic degradation of the composite in the oral environment when applied at low concentrations.^16,17^ However, this important molecule does not have any therapeutic properties, such as antimicrobial capacity, and the higher its concentration, the more susceptible the composite is to degradation. Therefore, it seems reasonable to use ionic liquids, which can show antibacterial activity, to create a novel silane, especially if this material could overcome the issue of composites’ degradation when used at higher concentrations.

Here, we synthesized an ionic liquid-based silane and used it to functionalize silicon dioxide (SiO_2_) nanoparticles, which were incorporated into an experimental resin composite. To tune the optimal concentration of the novel silane, SiO_2_ nanoparticles were functionalized with 1 to 10 wt % of ionic liquid-based silane previously their addition into the resin. The final composites were evaluated for their physical, chemical, and biological properties.

## 2 Materials and Methods

### 2.1 Synthesis of Ionic Liquid-Based Silane

**Figure 1:**
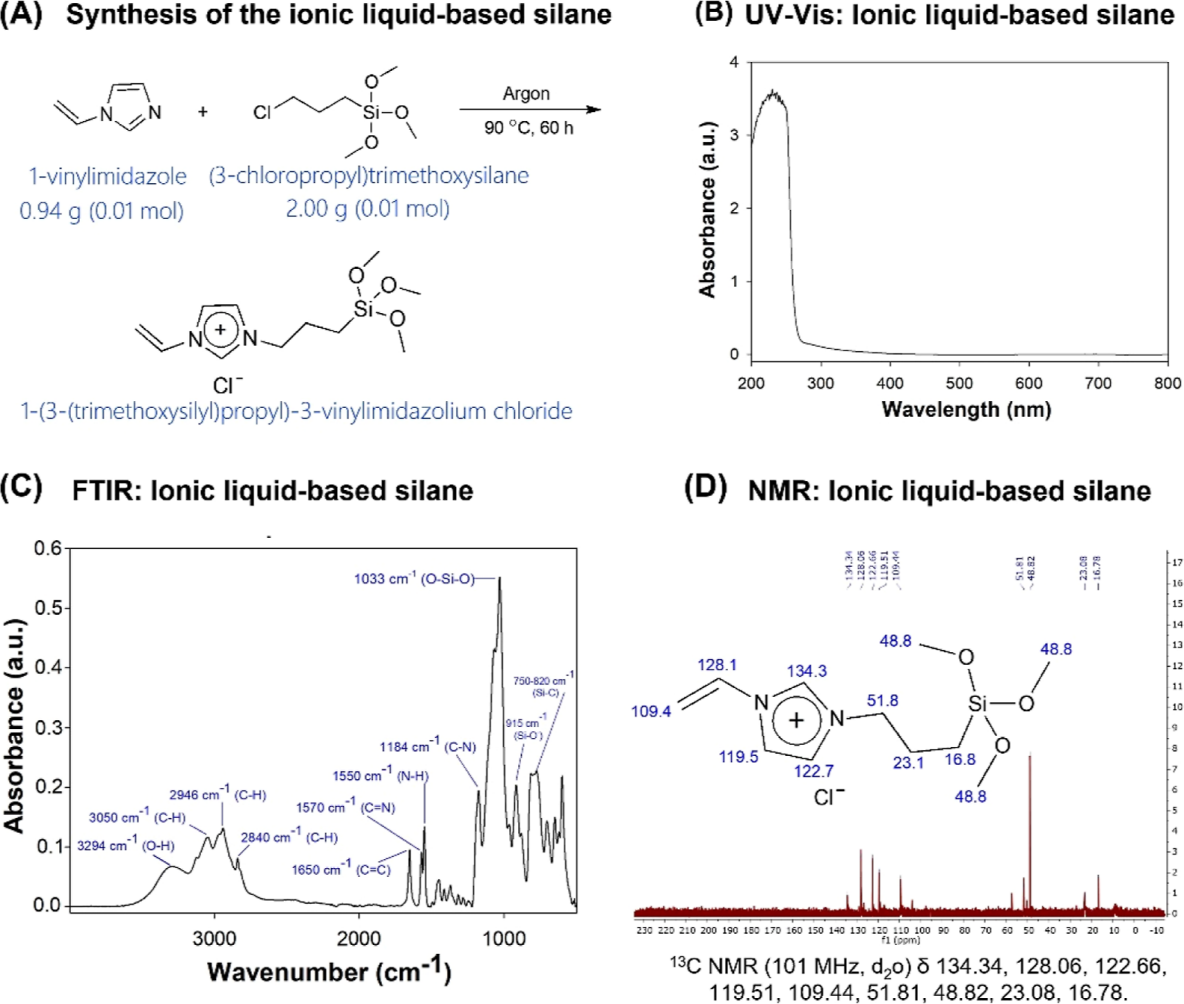
Ionic liquid-based silane synthesis and characterization. (A) Schematic representation of the ionic liquid-based silane synthesis. (B) UV–vis spectrum shows a band between 200 and 280 nm. (C) ATR-FTIR spectrum displays the characteristic peaks of the synthesized silane. (D) ^13^C NMR spectrum indicates the characteristic peaks of the synthesized silane, evidencing the suggested molecular structure by the presence of signals related to the imidazolium ring, the carbon–carbon double chain, and the aliphatic chain.

The ionic liquid-based silane was synthesized according to a previous study.^18^ In this solvent-free procedure, (3-chloropropyl)trimethoxysilane reacts with 1-vinylimidazole to produce the desired compound 1-(3-(trimethoxysilyl)propyl)-3-vinylimidazolium chloride (Figure 1A). First, 2.00 g (0.01 mol) of (3-chloropropyl)trimethoxysilane was mixed with 0.94 g (0.01 mol) of 1-vinylimidazole in a stainless steel autoclave reactor. This mixture was stirred for 60 h at 90 °C under argon. The mixture was cooled, washed with ether three times, and dried in a vacuum. The residual volatile substances were extracted under a low pressure. The reaction produced an oil, and the yield was 90%.

### 2.2 FTIR, UV–Vis, and NMR Analysis of the Ionic Liquid-Based Silane

The ionic liquid-based silane was chemically characterized by Fourier transform infrared (FTIR) spectroscopy (FTIR, Vertex 70, Bruker Optics, Ettlingen, Germany). The material was placed on the attenuated total reflectance (ATR) device and analyzed from 400 to 4000 cm^–1^, 4 cm^–1^ of resolution, and 32 scans. The synthesized material was also analyzed via ultraviolet–visible spectroscopy (UV–vis) with a Shimadzu UV–vis Spectrometer UV-2450 and a light wavelength range between 200 and 800 nm. Then, silane was studied via nuclear magnetic resonance (NMR) spectroscopy. ^1^H (400 MHz) and ^13^C (101 MHz) NMR spectra were acquired (Gemini 2000 NMR Spectrometer, Varian, Paolo Alto, CA, USA) with 20 mg of silane dissolved in deuterated chloroform. The chemical shifts (δ) were related in parts per million (ppm) concerning the internal standard (tetramethylsilane).

### 2.3 Silanization of SiO_2_ with Ionic Liquid-Based Silane

**Figure 2:**
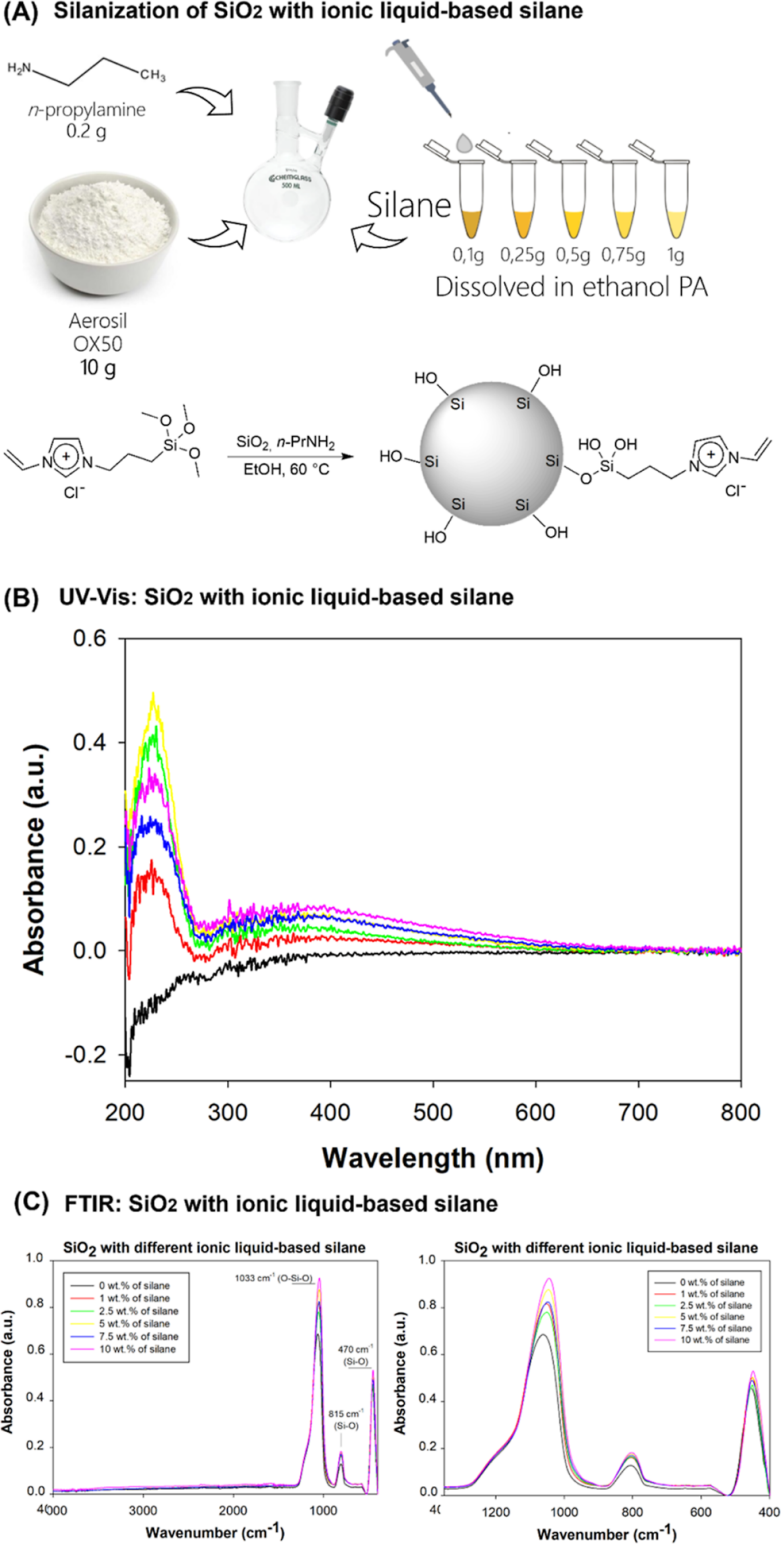
Silanization and characterization of SiO_2_ with different concentrations of ionic liquid-based silane. (A) Schematic representation of the silanization of SiO_2_ nanoparticles with the ionic liquid-based silane. (B) UV–vis analysis of the SiO_2_ modified with different concentrations of the ionic liquid-based silane. The group of SiO_2_ without the ionic liquid-based silane showed negative absorbance values or zero. The groups with 1 to 10 wt % of the silane showed absorbance values similar to the UV–vis characterization of the ionic liquid-based silane. (C) ATR-FTIR analysis of the SiO_2_ modified with different concentrations of the ionic liquid-based silane. The image on the left shows spectra from 400 to 4000 cm^–1^. The image on the right highlights the spectra displaying the results from 400 to 1350 cm^–1^. The higher the concentration of silane, the higher the absorbance peaks related to O–Si–O, Si–O, and Si–O.

The silanization of silicon dioxide nanoparticles (SiO_2_, Aerosil OX50, Lot no. 159031545) was based on a previous study.^19^ The functionalization of silica with the ionic liquid-based silane is shown in Figure 2A. First, 10 g of SiO_2_ was weighted in a 500 mL flask, in which 0.2 g of *n*-propylamine (Sigma-Aldrich Lot no. MKCK7066) was added. The ionic liquid-based silane was weighted (0.1, 0.25, 0.5, 0.75, or 1 g) in Eppendorf tubes. Ethanol PA (ACS Reatec, Lot no. 004404) was added to the Eppendorf tubes to dissolve the silane, which was mixed with the SiO_2_ and *n*-propylamine. More ethanol was added to the flask, totaling 200 mL of this solvent. A magnetic bar was added to the flask, and the mixture was stirred at room temperature for 30 min. Then the mixture was stirred for a further 30 min at 60 °C. After this period, the solvent was evaporated in a rotary evaporator, totaling about 40 min in a water bath at 60 °C and pressure of 212 mbar. The vials were then kept in a vacuum oven at 95 °C for 2 days. At the end of this process, SiO_2_ with 1, 2.5, 5, 7.5, and 10 wt % of ionic liquid-based silane was obtained.

### 2.4 FTIR, UV–Vis, and NMR Analysis of Functionalized SiO_2_ with Different Concentrations of the Ionic Liquid-Based Silane

SiO_2_ with different concentrations of ionic liquid-based silane (unfunctionalized, 0 wt %; functionalized, 1–10 wt %) was analyzed for FTIR-ATR. For this purpose, the powder (*n* = 1)^20^ was pressed against the ATR with a glass, and the samples were analyzed from 400 to 4000 cm^–1^, 4 cm^–1^ of resolution, and 32 scans. The powder (*n* = 1)^20^ was analyzed via ultraviolet–visible spectroscopy (UV–vis) with a Shimadzu UV–vis Spectrometer UV-2450 in a diffuse reflectance mode using an integrated sphere accessory and a light wavelength range between 200 and 800 nm. Solid-state ^13^C and ^29^Si NMR analyses of the SiO_2_ with and without the silane were performed on Agilent DD2 500 MHz equipment (for all samples, 10 kHz rotation). To achieve the analyses, we used an 11.7 T field and 500 MHz for ^1^H. For the ^13^C analysis, a pulse sequence of cross-polarization was used with PW90 of 2.75 μs, D1 of 5 s, and contact time of 7 ms. For the ^13^C analysis, a frequency of 125.7 MHz was used. For the ^29^Si analysis, a pulse sequence of cross-polarization was used with PW90 of 2.8 μs, D1 of 5 s, and contact time of 7 ms. Finally, for the ^29^Si analysis, a frequency of 99.3 MHz was used.

### 2.5 TGA of Functionalized SiO_2_ with Different Concentrations of Ionic Liquid-Based Silane

SiO_2_ (4 mg per group, *n* = 1) with varying concentrations of ionic liquid-based silane was analyzed for their thermogravimetric profile with an SDT Q600 device (TA Instruments, New Castle, Delaware, USA). 10 mg of each group was examined under a nitrogen atmosphere (100 mL/min) at 10 °C/min from room temperature up to 800 °C. TGA and derivative thermogravimetric (DTG) curves were obtained with Advantage Software v5.5.20 (Universal Analysis, TA Instrument, New Castle, Delaware, USA) and Origin 9.0 Software (OriginLab, Northampton, Massachusetts, USA).

### 2.6 Morphology Analysis of Functionalized SiO_2_ with Different Concentrations of Ionic Liquid-Based Silane

The morphology of SiO_2_ with different concentrations of the ionic liquid-based silane was analyzed via scanning electron microscopy (SEM) and transmission electron microscopy (TEM). The powders were fixed on metallic stubs with conductive carbon adhesive tapes for SEM analysis. Then, the samples were coated with a gold sputter (layer of 15–25 nm) (SCD 050, Baltec, Vaduz, Liechtenstein). The images were acquired by SEM (JSM-56060, Jeol, Akishima, Tokyo, Japan) with 10 kV and a magnification of 20,000×. For TEM, the powders were dispersed in water, and five drops of 4 μL each were applied onto a 200-mesh carbon-coated copper grid (SPI, West Chester, Pennsylvania, USA) and left to dry at room temperature. The images were acquired in a JEOL JEM-1011 microscope (JEOL USA, Inc., Peabody, MA, USA) at 100 kV and magnification of 20,000×.

### 2.7 Formulation of Experimental Resin Composites

The experimental resin composite was formulated by mixing 50 wt % of Bisphenol A glycol dimethacrylate (BisGMA, Aldrich Chemical Co., St. Louis, Missouri, USA) and 50 wt % of triethylene glycol dimethacrylate (TEGDMA, Aldrich Chemical Co., St. Louis, Missouri, USA).^19^ The monomers were hand-mixed for 5 min, sonicated for 180 s, and remixed for 5 min^13^ A photoinitiator/co/initiator system was added to the mixture: camphorquinone (Aldrich Chemical Co., St. Louis, Missouri, USA), at 1 mol %, and ethyl 4-dimethylaminobenzoate (Aldrich Chemical Co., St. Louis, Missouri, USA), at 1 mol %. Butylated hydroxytoluene (Aldrich Chemical Co., St. Louis, Missouri, USA) was also added at 0.1 wt % as a polymerization inhibitor.^13^ The mixture was hand-mixed, sonicated, and hand-mixed again and kept at room temperature for 24 h.

The silanized SiO_2_ was added to the resin at 60 wt %,^19^ totalizing five groups: G_1%_, G_2.5%_, G_5%_, G_7.5%_, and G_10%,_ according to the quantity of ionic liquid-based silane used in the silanization process. Furthermore, one group with 60 wt % of nonsilanized SiO_2_ was added as a control group (G_CTRL_). Initially, the composite resins were hand-mixed, sonicated in the water bath and hand-mixed again. Then, each mixture was sonicated with an ultrasound-guided needle with a frequency of 20 kHz, 10% amplitude, for 10 s, followed by 20 s without sonication. This process was repeated for 2 min. The resin composites were placed in a 50 mL vial, which remained immersed in an ice-containing flask throughout the sonication.

### 2.8 TGA of Resin Composites

The thermogravimetric degradation profile was analyzed by using the polymerized resin composites. One sample (*n* = 1)^21^ per group was prepared (4 mm diameter, 1 mm thickness) with 20 s of photoactivation on each side (top and bottom). For photoactivation, a light-emitted diode device (VALO Cordless, Ultradent Products, South Jordan, Utah, USA) with a power of 1000 mW/cm^2^ was used throughout this study. With the aid of a scalpel blade, approximately 10 mg of each sample was obtained to be analyzed. The SDT Q600 device was used with nitrogen gas (100 mL/min) at 10 °C/min from room temperature to 800 °C. TGA and DTG curves were analyzed with Advantage Software v5.5.20 and Origin 9.0 Software.

### 2.9 Degree of Conversion

The degree of conversion (DC) was analyzed with five samples per group (*n* = 5)^21−23^ in a Fourier-transform infrared spectrometer (FTIR, Vertex 70, Bruker Optics, Ettlingen, Germany) with an attenuated total reflectance device (ATR, Platinum ATR-QL, Bruker Optics, Ettlingen, Germany). Each uncured resin composite sample was placed on the ATR with the aid of a polyvinylsiloxane mold with a 1 mm thickness. The samples were placed on the mold, and a polyester strip was placed on top of each one to standardize their thickness. One spectrum per sample was acquired before and after 20 s of photoactivation. The light-curing unit tip was positioned in contact with the strip. Opus software (Opus 6.5, Bruker Optics, Ettlingen, Germany) with Blackman-Harris 3-Term apodization from 4000 to 400 cm^–1^ was used with 4 cm^–1^ resolution. The DC was calculated using the height of two peaks: 1640 cm^–1^ (related to the aliphatic carbon–carbon double bond stretching vibration) and 1610 cm^–1^ (associated with the aromatic carbon–carbon double bond stretching vibration). The following equation was used:^22^
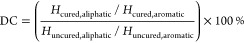
1where *H*_cured,aliphatic_ and *H*_cured,aromatic_ are the peak heights of cured aliphatic and aromatic C=C bonds, respectively, and *H*_uncured,aliphatic_ and *H*_uncured,aromatic_ are the peak heights of uncured aliphatic and aromatic C=C bonds, respectively.

The samples were removed from the ATR and stored in 1 mL of distilled water at 37 °C. After 2 months, the cured samples were analyzed again via FTIR-ATR. One spectrum per polymeric sample was obtained, and the heights of the 1640 and 1610 cm^–1^ peaks were measured again. The DC % after two months (*n* = 5) was calculated using the initially estimated heights for the uncured samples and the measurements acquired from those samples that were stored in distilled water.

### 2.10 Softening in Solvent

Five samples per group (*n* = 5)^21,23,24^ were prepared with 1 mm thickness and 4 mm diameter with 20 s of photoactivation on each side (top and bottom). The samples were kept in distilled water at 37 °C for 24 h. The samples were embedded in self-curing acrylic resin and polished with silicon carbide sandpapers (600 to 2000 grit), followed by a felt disc with alumina suspension. The samples were washed with distilled water in a sonicator for 380 s. After 24 h, the samples were analyzed for initial Knoop hardness (KHN1) with five indentations (10 g/5 s)^25^ per sample (HMV 2; Shimadzu, Tokyo, Japan). They were stored in an ethanolic solution (70% ethanol and 30% distilled water), washed under running conventional water, and reanalyzed (KHN2). The difference between KHN1 and KHN2 was calculated as percentage (ΔKHN %) for each sample and group.

### 2.11 Flexural Strength

The flexural strength was evaluated according to ISO 4049.^26^ Ten samples per group (*n* = 10)^25,27^ were prepared with a rectangular shape (25 mm × 2 mm × 2 mm). The uncured resin was placed into a metallic mold with a polyester strip on top and the bottom. The photoactivation was performed in four windows for 20 s on the bottom and top of the samples. The samples were stored in distilled water at 37 °C for 24 h for immediate analysis, and the other 10 samples (*n* = 10) were prepared and stored for 6 months at the sample conditions for the long-term test. The flexural strength was established with a three-point test at 0.75 mm/min in a universal mechanical testing machine (EZ-SX, Shimadzu, Kyoto, Japan) until the samples’ fracture. The flexural strength was calculated according to eq 2
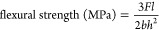
2where “*F*” is the maximum load applied on the sample, “*l*” is the distance (mm) between the supports ±0.01 mm, “*b*” is the width (mm) of the sample specimen immediately before testing, and “*h*” is the height (mm) of the sample. The values were measured with a digital caliper before testing.

### 2.12 Ultimate Tensile Strength

Ten samples per group (*n* = 10)^23,28,29^ were prepared for the ultimate tensile strength (UTS) test. The uncured samples were placed in a metallic mold with an hourglass shape (8.0 mm long, 2.0 mm wide, 1.0 mm thickness, 1 mm^2^ cross-sectional area), and a polyester strip was positioned on the top and bottom of the resin composite. Each sample was photoactivated for 20 s on each side (top and bottom) with the tip of the light-curing unit in contact with the polyester strip. The samples were stored in distilled water at 37 °C for 24 h. The constriction area of each sample was measured with a digital caliper (Mitutoyo, Kawasaki, Kanagawa, Japan), and the samples were fixed with cyanoacrylate in the metallic jigs. The samples were tensile until fracture (microtensile strength at 1 mm/min) by a universal mechanical testing machine (EZ-SX Series, Shimadzu, Kyoto, Japan) to acquire values in Newton (N). The value of each sample was divided by the results in Newton to be expressed in megapascal (MPa) (eq 3)^22^
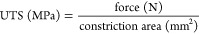
3

Another 10 samples per group (*n* = 10) were prepared and stored for 6 months in distilled water in an incubator at 37 °C to analyze the long-term UTS. The samples were measured, glued on the metallic jigs, and tested for UTS as described above.

### 2.13 Cytotoxicity against Human Gingival Fibroblasts

Gingival fibroblasts were obtained from human gingiva after the patient signed informed consent (local Ethics Committee no. 03294318.0.0000.5347). Gingival tissues were collected from surgical aesthetic procedures. Fibroblasts were isolated through the explant method, and the cells were used at the 5° passage. First, the cells were grown in Dulbecco’s modified Eagle’s medium (DMEM, Thermo Scientific, Waltham, MA, USA) with 10% fetal bovine serum (Thermo Scientific, 12657FBS), 5 mM Hepes (Thermo Scientific, 15630080), 3.7 g of sodium bicarbonate (Sigma-Aldrich, S5761), 100 U/mL penicillin, and 100 mg/mL streptomycin (Thermo Scientific, 15240062). The tubes were maintained in an incubator (37 °C, 5% CO_2_), and the cells were monitored daily in an inverted-phase microscope (Axiocam 105 color, Carl Zeiss Ltd., Oberkochen, Baden-Württemberg, Germany). The culture medium was changed every 2 or 3 days to achieve confluent cultures in 75 cm^2^ flasks.

Five disc-shaped samples per group (*n* = 5)^23,30,31^ were prepared with photoactivation for 20 s on each side to perform the SRB analysis. The samples were stored in distilled water for 24 h at 37 °C and then sterilized with a hydrogen peroxide plasma. On the first day of the experiment, each sample was immersed in 1 mL of DMEM for 24 h at 37 °C to obtain the eluates. Moreover, the gingival fibroblasts were seeded in the wells of 96-well microplates at 5 × 10^3^ per well in 100 μL of DMEM media. The microplates were stored in an incubator for 24 h at 37 °C and 5% of CO_2_. On the next day, 100 μL of eluate from each sample was added to the wells in triplicate. Five wells were used as negative controls that remained without the eluates, adding 100 μL of fresh DMEM. After 72 h of incubation at 37 °C and 5% CO_2_, the cells were fixed at the bottom of the wells with 50 μL of trichloroacetic acid (10%) for 1 h at 4 °C. The microplates were gently washed six times with running distilled water for 30 s and kept at room temperature until dry. After this period, 50 μL of SRB (Sigma-Aldrich, 3520-42-1) at 0.4% acetic acid was added to each well. The microplates were incubated for 30 min at room temperature, washed with acetic acid at 1% four times, and then allowed to dry again at room temperature. Finally, 100 μL of Trizma solution at 10 mM was added to each well, and the microplates were incubated for 1 h. The optical density of each well was analyzed by reading the absorbance with a spectrometer at 560 nm (Thermo Fisher Scientific, Thermo Scientific Multiskan GO, Waltham, MA, USA). The cell viability was expressed in percentages compared to wells without eluates (negative control) as 100%.

### 2.14 Biofilm Model with *Streptococcus mutans*

*S. mutans* UA159 was cultured overnight for 18 h at 5% CO_2_ and 37 °C in brain heart infusion (BHI) broth (Sigma-Aldrich). To prepare the inoculum, the culture was adjusted to an optical density at 600 nm (OD_600_) of 0.9 in BHI broth supplemented with 1 wt % of sucrose. The inoculum (1.5 mL) was added to each well of a 24-well culture plate. The samples were placed at the bottom of each well and incubated for 24 h at 5% CO_2_ and 37 °C. The medium was replaced after 24 h for a fresh BHI broth (1.5 mL/well) with 1 wt % of sucrose. The samples were incubated with the fresh medium for more than 24 h at 5% CO_2_ and 37 °C, totaling 48 h of biofilm formation.

### 2.15 Fluorescence Microscopy Staining

After the 48 h biofilm formation, the samples were removed from the plate and gently washed (2×) with PBS buffer (pH = 7.2). The samples were placed on absorbent paper and stained with BacLight live/dead kit solution (Molecular Probes, Eugene, OR, USA). SYTO 9 and propidium iodide (0.1% propidium iodide and 0.1% Syto 9 in 0.85% NaCl) were mixed. The samples were incubated at the prepared solution for 15 min in the dark (23 °C). An inverted epifluorescence microscope (Eclipse TE2000-S, Nikon, Melville, NY, USA) was used at 483 nm to excite SYTO9 (staining of live bacteria) and at 535 nm to excite propidium iodide (staining dead bacteria). The fluorescence of live bacteria was acquired at 503 nm (green) and that of dead bacteria at 617 nm (red).

### 2.16 Colony Forming Units Counting Assay

After 48 h, each sample (*n* = 6) was transferred to a glass vial containing 1 mL of cysteine peptide water and three 1 mm glass beads for the vortex-sonication-vortex technique. This protocol is performed by vortex-mixing for 10 s and sonication for 5 min (Branson 3510-DTH Ultrasonic Cleaner) and vortexing again for biofilm dislodging. Aliquots of bacterial suspension were collected and serially diluted from 10^–1^ to 10^–6^ via the drop-plate method. Three drops (10 μL each) from each dilution were plated on Brain Heart Infusion agar and incubated at 5% CO_2_ and 37 °C. After 48 h, the plates were removed, and colonies were counted where the dilution contained 30–300 colonies per 10 μL drop. The results were calculated based on the number of CFU and the dilution factor. The values were log_10_ transformed and expressed as cfu/mL.

### 2.17 Statistical Analysis

The ionic liquid-based silane characterization, SiO_2_ characterization, SEM, TEM, and microscopic fluorescence images were descriptively analyzed. The data from resin composites evaluation were analyzed (SigmaPlot, Systat Software, version 12.0, San Jose, CA, USA) for normality and homoscedasticity with Shapiro–Wilk and Levene’s test, respectively. Two-way ANOVA followed by Tukey posthoc was applied to compare groups for FS and UTS considering the following: factor 1, the concentration of ionic liquid-based silane; factor 2, time of storage in water. One-way ANOVA followed by Tukey posthoc was applied to compare the resin composite for the immediate DC %, long-term DC %, KHN1, ΔKHN %, cytotoxicity, and antibacterial activity via CFU assay. The differences from KHN1 to KHN2 and immediate to long-term DC % within each group were analyzed via paired *t*-test. A significance level of 0.05 was used in all of the tests.

## 3 Results and Discussion

In this study, an imidazolium ionic liquid was used in the synthesis of an organosilane due to the imidazole ring’s biological properties, such as the fungicidal effect and antibacterial activity.^8^ Moreover, we used an ionic liquid with only three carbons in the aliphatic chain for two reasons: reduction of possible cytotoxic consequences^32^ and possible plasticizing effect, maintaining a chain size equal to the commercially used silane (3-(trimethoxysilyl)propyl methacrylate (MPTS).^13^ As to the anion choice, a simple halide anion (Cl^–^) was used to decrease possible cytotoxicity. The characterization of the synthesized ionic liquid-based silane showed that we synthesized the intended material.

The results of the characterization of the ionic liquid-based silane via UV–vis, FTIR, and NMR spectroscopies are shown in Figure 1. In the UV–vis spectroscopy (Figure 1B), the absorbance band of the ionic liquid-based silane showed a peak at 230 nm, in a range from 270 to 200 nm, which is assigned to the π → π* transition. The FTIR analysis (Figure 1C) showed a characteristic spectrum of the synthesized silane. The most intense FTIR absorbance peaks were indicated with their respective chemical groups in the spectrum. In particular, the peaks characteristic of the imidazolium ring at 1184, 1550,^33^ and 1570 cm^–1^^34^ can be assigned to the C–N, N–H, and C=N bonds, respectively. The peaks referring to chemical bonds with silicon are indicated at 1033,^35^ 915,^36^ and 750–820 cm^–1^,^37^ which are referred to O–Si–O, Si–O, and Si–C bonds, respectively. The band indicating the presence of the aliphatic C=C is observed at 1650 cm^–1^,^22^ and the aliphatic C–H bonds are presented at 2840–3050 cm^–1^. ^13^C NMR spectroscopy (Figure 1D) allowed for the elucidation of the chemical structure of the ionic liquid-based silane, where the observed peaks confirmed the expected chemical structure and the success of the synthetic procedure.

The development of polymerizable ionic liquids is essential to boost their application in dentistry. The analyses of SiO_2_ without and with the synthesized silane showed the presence of the ionic liquid-based silane in the powder (UV–vis spectra) and the successful silanization (NMR spectra of powders). The results of UV–vis spectroscopies of functionalized SiO_2_ with different concentrations of the ionic liquid-based silane are shown in Figure 2B. The absorbance band showed negative values or 0.0 nm for SiO_2_ without the silane. Groups with 1 to 10 wt % of the ionic liquid-based silane showed a strong peak at 230 nm (band range from 270 to 200 nm) related to the π → π* transition of the imidazole ring and a broad but weak absorption band between 270 and 600 nm probably from the SiO_2_.

The FTIR analysis of the functionalized SiO_2_ with different silane concentrations showed three peaks (Figure 2C): 1033 cm^–1^ related to O–Si–O, 815, and 470 cm^–1^ related to Si–O. The zoomed-in image shown in Figure 2C is the spectra in the region from 400 to 1350 cm^–1^. It is possible to observe that increasing the amount of ionic liquid-based silane from 0 to 10 wt % increases the absorbance of these peaks.

**Figure 3:**
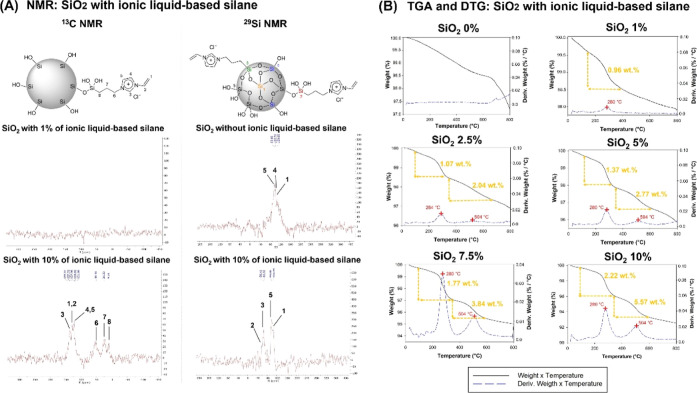
NMR and TGA of SiO_2_ modified with different concentrations of the ionic liquid-based silane. (A) NMR spectra. On the left, the schematic illustration is on the left of one particle of SiO_2_ modified with ionic liquid-based silane. The scheme suggests how the silane interacts with the SiO_2_ particle considering the results from the ^13^C NMR technique. The spectra on the right side are related to functionalized SiO_2_ with 1 and 10 wt % of ionic liquid-based silane. On the right, the schematic illustration of the silicon species of the ionic liquid (2 and 3) and the silica network (1, 4, and 5). The scheme suggests how the ionic liquid-based silane is bonded in the SiO_2_ particle considering the ^29^Si NMR technique. The spectra on the right side are related to SiO_2_ with 0 and 10 wt % of ionic liquid-based silane. (B) TGA and DTG results. The higher the silane concentration, the higher the DTG peaks at 280 and 504 °C.

The ^13^C NMR technique was used to clarify the characteristic signals of the ionic liquid-based silane bonded to the silica. Figure 3A depicts a schematic illustration of the molecular structure of the ionic liquid bonded to SiO_2_ as suggested by the ^13^C NMR technique. The spectra of the functionalized SiO_2_ with 1 and 10 wt % of ionic liquid-based silane are also indicated. Table S1 shows the signals of ^13^C NMR. The carbon-related signal (8) appeared at approximately 8 to 9 ppm. Peaks at 119 and 125 ppm were assigned to carbons (4) and (5), and the signal at 135–136 ppm was assigned to carbon (3) of the imidazolium cation. The carbon signal (6) appeared at 50–51 ppm. The central carbon (7) of the propyl chain, which connects the imidazolium ring to the Si atom, was observed between 22 and 24 ppm. The vinyl carbons (1) and (2) attached to the imidazolium ring appeared between 128 and 129 ppm.

The ^29^Si NMR technique investigated how the ionic liquid was bonded to the silica network structure. Figure 3A also depicts a schematic illustration of the molecular structure of the ionic liquid bonded to SiO_2_ as suggested by the ^29^Si NMR technique. The spectra of the lowest (0%) and highest (10%) concentrations of ionic liquid-based silane used to modify SiO_2_ are also indicated. Table S2 shows the signals of ^29^Si NMR. The spectra exhibited signs at −99 ppm and between −102.8 and −100 ppm, which are attributed to species 5 and 4, ((HO)_2_Si(OSi)_2_) and (Si(OSi)_3_(OH)), respectively. The signal between −113.2 and −106.4 ppm is referred to as species 1, (Si(OSi)_4_). The presence of the signal between −60.6 and −66.8 ppm relative to substructure 3, (RSi(OSi)_3_), and the signal between 52.2 and 61.7 ppm relative to structure 2, R-Si(RO)_2_(OSi), indicates the existence of bonds between silica and ionic liquid molecules. These two facts suggest good condensation between the inorganic (SiO_2_ network) and organic (ionic liquid) parts. Figures S3 and S4 show the spectra from 2.5 to 7.5% of the ^13^C NMR technique and from 1 to 7.5% of the ^29^Si NMR technique, respectively.

The synthesized silane is a bifunctional molecule with silanol (Si–OH) and methacrylate groups on one side.^15^ The silane is hydrolyzed and subsequently undergoes a condensation reaction to act as a binding agent. In this process, the methoxy groups (–Si(OCH_3_)_3_) of the silane are hydrolyzed to produce reactive silanol groups (–Si(OH)_3_).^15^ These bind to the silanols on the surface of the SiO_2_ particle through the hydroxyl (OH), forming hydrogen bonds. The commercial silane MPTS also has hydrogen bridges between the carbonyl group (C=O) of the MPTS and the hydroxyl group of the SiO_2_ surface.^15^ In our case, there is no C=O, and NMR showed the interaction between silane and SiO_2_ only between the silane’s silanol and the particle’s hydroxyl, forming a Si–O–Si covalent bond through a reaction of condensation in which water is released.^15^ Although the FTIR of the particles did not show the presence of C=C (probably due to the low percentage of silane), it was evident in the silane’ characterization in the FTIR and NMR spectra and in the SiO_2_ description via NMR, suggesting that this functional group would be free to bond with the resin network.

Figure 3B shows the graphs of TGA and DTG of functionalized SiO_2_ with different concentrations of the ionic liquid-based silane. SiO_2_ with 0 wt % of silane showed a TGA curve with a weight loss of 2.81%, probably related to water removal. The powders with 1 to 5 wt % of ionic liquid-based silane revealed another weight-loss stage at 280 °C. The powders of SiO_2_ with 7.5 and 10 wt % also showed a third stage of weight loss at around 500 °C. These peaks at 280 and 504 °C are probably related to the degradation of the organic part of the ionic liquid-based silane, and they are more intense the higher the silane content. Therefore, these results from TGA analyses suggested their functionalization due to the difference in the percentage of degradation observed by TGA compared with the amount of silane mixed with SiO_2_ nanoparticles during the silanization process. The methoxy groups (–Si(OCH_3_)_3_) of the silane hydrolyze to form silanol groups (–Si(OH)_3_) during the silanization process, resulting in a mass decrease of the silane compound bonded to the silica compared with the initial quantity of silane mixed.

Figure S5 presents SEM and TEM images of functionalized SiO_2_ containing different concentrations of the ionic liquid-based silane. In SEM analysis, it is possible to observe a degree of particle aggregation in the group with 10 wt % of silane, while in the 0% group, the particles are less agglomerated. In the case of functionalized silica, the presence of the organic modifier generates a local particle aggregation. This fact was observed from 1 to 10 wt % of silane, but it was more pronounced from 5 to 10 wt % of silanes. On the other hand, differences among groups in the TEM images were not observed, suggesting that ionic liquid-based silane in the functionalized silica did not cause any significant morphological change compared to the silica without the modifier.

**Figure 4:**
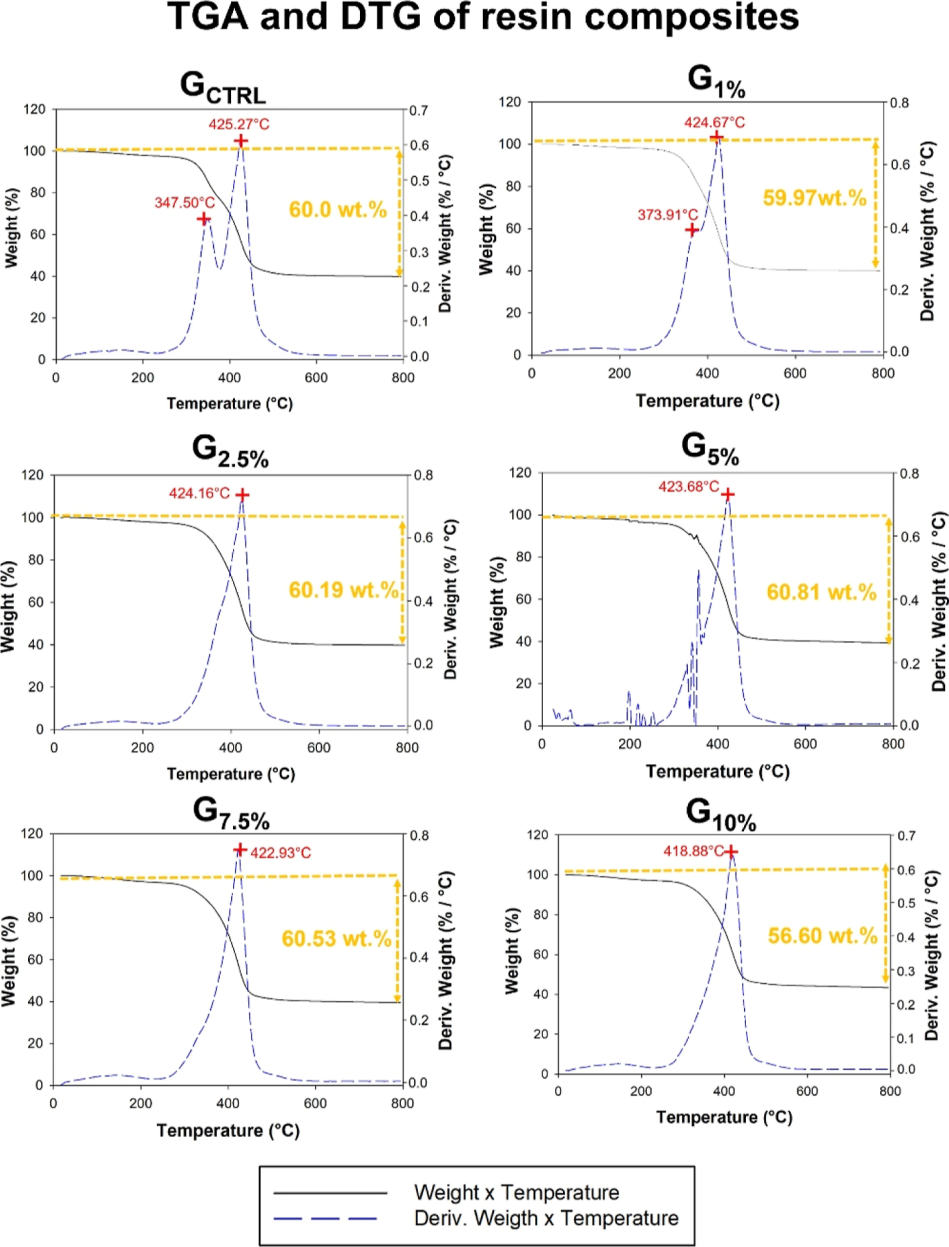
TGA of resin composites. The images show the thermogravimetric analysis of resin composites without ionic liquid up to 10 wt % of ionic liquid used as silane of SiO_2_. All groups showed a residue around 40 wt %, which was the final concentration of SiO_2_ in the formulation of all resin composite groups. The resin composite with a higher ionic liquid concentration showed higher thermal stability since it showed lower weight loss.

Figure 4 displays the results of TGA and DTG of the formulated resin composites. G_CTRL_ and G_1%_ showed two stages of weight loss at around 345 and 425 °C that are probably related to TEGDMA and BisGMA degradation, respectively. When at least 2.5 wt % of ionic liquid-based silane was used to silanize the filler, the peak of TEGDMA almost disappeared, changing to one single peak at around 420 °C. Practically no differences are observed in the decomposition temperature among G_2.5%_ and G_10%_.

**Figure 5:**
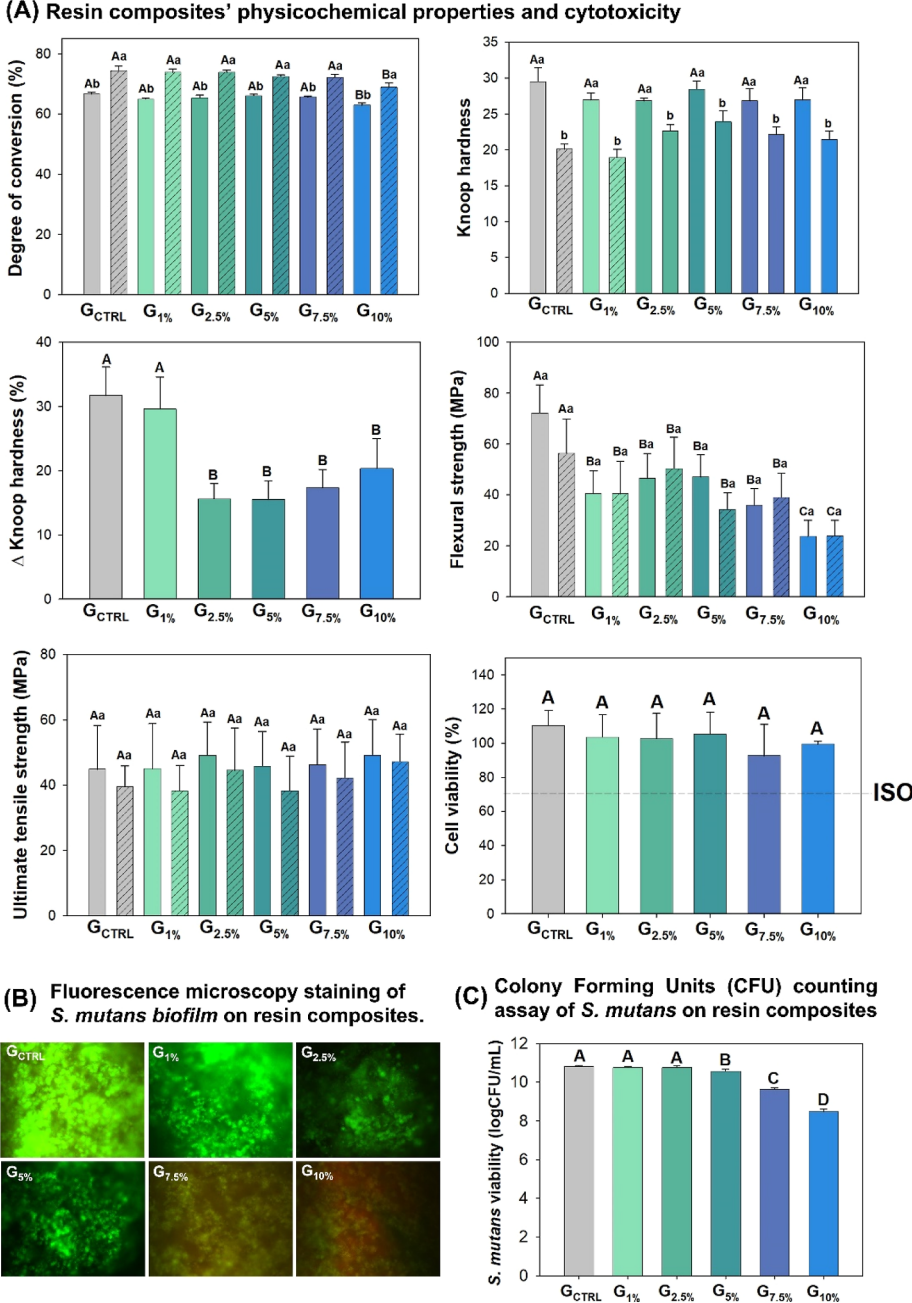
Physicochemical properties, cytotoxicity, and antibacterial activity of the formulated resin composites. (A) Image shows the graphs of degree of conversion (%), Knoop hardness, Knoop hardness variation (softening in solvent) (%), flexural strength (MPa), ultimate tensile strength (MPa), and human cell viability (%). The line in the graph of human cell viability indicates the threshold of 70% stabilized by ISO. Different capital letters indicate statistical differences among groups in the same test (*p* < 0.05). (B) Fluorescence microscopy images of *S. mutans* biofilms developed on top of the resin composites. The green staining of biofilms indicates that they are composed of bacteria without membrane damage. The red staining of biofilms suggests that they are composed of bacteria with membrane damage. (C) Image represents the CFU assay’s means and standard deviation values of *S. mutans*. Different capital letters indicate statistically significant differences among groups (*p* < 0.05).

The results of immediate DC %, long-term DC %, KHN1, KHN2, and ΔKHN % are presented in Figure 5A. The DC % ranged from 62.98 (±0.73)% for G_10%_ to 66.64 (±0.58)% to G_CTRL_ (*p* < 0.001). The groups with 0 to 7.5 wt % of the ionic liquid-based silane showed higher DC % than G_10%_ (*p* < 0.001), and there was no statistical difference among G_CTRL_, G_2.5%_, G_5%_, and G_7.5%_. All groups presented higher DC % after the storage of the samples for 2 months in distilled water at 37 °C (*p* < 0.05), with values ranging from 68.89 (±1.39)% for G_10%_ to 74.45 (±1.45)% for G_CTRL_ (*p* < 0.001). The group containing 10 wt % of the ionic liquid-based silane showed the lowest long-term DC % (*p* < 0.001).

A proper DC % can assist in achieving reliable mechanical properties and lead to lower solubility,^38^ decreased leaching compounds, and reduced cytotoxicity propensity.^39^ In the immediate analysis, G_10%_ showed a significantly lower DC % that might be related to the darkest color observed for this group. The different color of light-cured resins influences the light transmission through the material.^40^ The darker the resin, the less light is transmitted and the lower the DC %. After 2 months, all groups showed higher DC %. This outcome’s rationale is associated with the commonly delayed polymerization after photoactivation.^41−43^ Moreover, cross-linked dimethacrylates may have been lost over time, increasing the calculated value of DC %.^42,43^ After the storage in water, G_10%_ maintained a lower DC % compared to the other groups. However, all groups presented values compatible with commercial resin composites.^43^

The softening in solvent test showed no statistical differences among groups for KHN1 (*p* = 0.04), and all groups had lower KHN2 compared to KHN1 (*p* < 0.05) (Figure 5A). The ΔKHN % ranged from 15.50 (±2.97)% for G_5%_ to 31.74 (±4.40)% for G_CTRL_ (*p* < 0.001). The addition of at least 2.5 wt % of the ionic liquid-based silane led to less ΔKHN% compared to G_CTRL_ and G_1%_ (*p* < 0.001), without statistically significant differences from 2.5 to 10 wt % of silane addition (*p* > 0.05).

The resin composites containing SiO_2_ functionalized with ionic liquid-based silane showed lower FS than G_CTRL_ (*p* < 0.05) (Figure 5A). There was no statistically significant difference between immediate and long-term analyses within each group (*p* > 0.05). The UTS revealed no differences among groups regardless of the ionic liquid-based silane concentration (*p* > 0.05) and the time of storage (*p* > 0.05).

The resin matrix used in this study (50:50 BisGMA/TEGDMA) is a classical blend that was already tested in previous research.^19,42^ The same SiO_2_ nanoparticles were used in those studies, leading to the possibility of comparison with our results. Here, the FS decreased with ionic liquid-based silane addition, which was not observed in the UTS test. The FS test is more complex than the UTS. The FS occurs when tensile, compressive, and shear stresses coincide. On the other side, the UTS test induces only tensile strength. The FS may be more sensitive than the UTS to show differences in the behavior of materials. As previously suggested,^19^ the use of excessive content of silane (more than 2.5 or 5 wt %) can lead to their molecular arrangement in multilayers on SiO_2_ surfaces: the first layer is covalently bonded to the SiO_2_. In contrast, the second is linked to the first through hydrogen bonds, without direct bonding between the second layer and SiO_2_.^19^ The excessive layers of ionic liquid-based silane could be why the higher the concentration, the lower the FS. Here, we employed the same SiO_2_ nanoparticles (40 nm) as those utilized in prior studies^19,44^ to facilitate result comparison. However, for future investigations and potential market resin composite enhancements, the authors propose exploring alternative glass compositions to enhance the FS of these composites.

It is interesting to observe that in the FS and UTS, aging did not influence in the behaviors among groups. However, in the softening in solvent analysis, the groups with at least 2.5 wt % of silane showed greater resistance against the alcoholic solution. This could be explained by the difference in the solubility parameters of the materials tested. The smaller the difference in the solubility parameter between the solvent and the polymer, the more the solvent can penetrate, softening and degrading the resin network.^44^ The alcohol solubility parameter is closer to the solubility parameter of resins composed of BisGMA and TEGDMA compared to the water parameter.^44^ Therefore, the softening in solvent test is a higher challenge for the composites than immersion of the samples in distilled water. If the samples were kept longer in water in the FS and UTS, it is possible that the result found in the softening in solvent could also be observed, with a protective effect of silane against the liquid medium.

The results mentioned above agree with a previous study, in which the concentration of at least 2 wt % of MPTS reduced water sorption and stabilized the Knoop hardness after 4 months of storage in distilled water, protecting the resin composite against degradation.^42^ Sideridou and Karabela^19^ also found that 2.5 and 5 wt % of MPTS protected the composite against sorption in water, while 2.5 wt % protected against sorption in an ethanolic solution (ethanol/water 75 vol %). 2.5 wt % of MPTS also reduced the solubility in water or in the ethanolic solution.^19^ In contrast with our findings, Sideridou and Karabela observed that higher concentrations of MPTS (from 5 to 10 wt %, depending on the test) jeopardized the stability of resin composites, increasing the sorption and solubility.^19^ They suggested that the multilayer organization of MPTS surrounding the SiO_2_ was the reason for the higher vulnerability to hydrolysis for such groups.^19^ However, we did not observe differences from G_2.5%_ to G_10%_ for ΔKHN %, since all groups with at least 2.5% reduced the softening in solvent. The differences between MPTS and the synthesized ionic liquid-based silane, especially regarding the absence of carbonyl groups in the experimental silane, may have favored the hydrolytic behavior of this last one compared to MPTS. Interestingly, from 2.5 wt % of silane, the TGA began to indicate the union between the two peaks of 347.50 and 425.27 that appeared in the G_CTRL_. This effect was observed in a previous study, in which an ionic liquid was incorporated at different concentrations in a composite resin for orthodontics.^21^ It was suggested that replacing two peaks with one peak could result in the formation of a more homogeneous resin matrix.^21^

The fluorescence microscopy with Live/Dead dye revealed more areas stained in red from 5 wt % of ionic liquid-based silane (Figure 5B). According to the CFU assay, the higher the concentration of ionic liquid-based silane, the lower the viability of *S. mutans* biofilm. From 5 wt % of the experimental silane, there was a statistically significant lower log cfu/mL compared to G_CTRL_, G_1%_, and G_2.5%_ (*p* < 0.05). There were no differences from 0 to 2.5 wt % (*p* > 0.05).

The antimicrobial activity of ionic liquids is one of the reasons why they have been studied as active agents in drugs.^10^ Currently, about 50% of the available drugs are administered as salts, which usually have disadvantages such as a high melting temperature (negatively influencing drug processing) and spontaneous polymorphic transformation.^10^ These factors increase processing costs, reduce drug safety, and reduce the drug shelf life. The use of salts in liquid form, such as ionic liquids, could avoid such issues, especially the polymorphism problems associated with solids.^10^ Furthermore, the possibility of changing the cation, anion, and chain length of ionic liquids causes them to be innovative antimicrobial strategies capable of providing the chemical diversity necessary to design new drugs to overcome microbial resistance issues.^8^ Even though the fluorescence microscopy showed membrane defects in bacteria from 7.5% of silane concentration, the gold standard CFU analysis showed that the polymers presented antibacterial activity from 5 to 10 wt % of silane. These values correspond from 2.5 to 5 wt % of silane in the resin composite. The antibacterial activity of ionic liquids has not been fully elucidated. However, due to structural analogies with quaternary ammonium compounds, it is suggested that the antibacterial activity of ionic liquids occurs similarly to that of quaternary ammonium compounds. Two factors are considered most relevant for this: lipophilicity and the predominance of positive electric charge. It is known that the longer the alkyl chain of the ionic liquid, the greater its lipophilicity, increasing the chances of interaction with bacterial membranes and walls, thereby enhancing the antibacterial activity. Additionally, the positive charge of the large cationic cores of ionic liquids is an important factor in providing antibacterial activity, as most of the electric charges on the surface of bacteria are negative, attracting positively charged molecules. These organic salts likely come into contact with bacteria, causing lysis of their surface, leading to cellular death.^8,13^

Finally, the SRB method tested the experimental resin composites for possible cytotoxicity. There were no differences among the resin composites in the cytotoxicity analysis, regardless of the ionic liquid-based silane concentration (*p* > 0.05). All groups showed values of cell viability higher than 70%, suggesting that the materials are not cytotoxic according to ISO 10993-5^45^ (Figure 5A). Previous studies with imidazole-based ionic liquids added purely into dental resins,^21^ into microcapsules,^31^ and covering quantum dots^13^ showed similar results without affecting human cells. The short alkyl chain and the simple anion used in the synthesis probably aided in reducing possible cytotoxic effects.

It was beyond the scope of this study to compare commercial silane with the ionic liquid-based silane. However, future studies should investigate whether the physicochemical properties are altered compared with commercial ones. Since the experimental silane does not have C=O, a group potentially degraded by water over time, the ionic liquid-based silane may have less degradation than MPTS. However, these properties probably depend on the anion used. In addition to comparing it with MPTS, more hydrophobic anions (such as NTf_2_) could be analyzed to increase the antibacterial activity possibly.

While the existing literature has explored other formulations of cationic nanoparticles (NPs) with antibacterial properties, our work transcends the conventional approach by integrating an ionic liquid (IL) into the resin formulation, replacing traditional organosilanes with an ionic liquid for the first time. The novelty of our approach lies in the application of ionic liquids, which offer multifaceted advantages beyond the antibacterial efficacy. These compounds have remarkable versatility. Through anion, aliphatic chain size, and cation changes, we can bestow a diverse array of biological properties on the resin. By harnessing the potential of these organic salts, we lay the groundwork for future composite resins endowed with specific functionalities tailored to meet diverse clinical needs. In essence, our work represents a pioneering step toward the development of composite resins that transcend conventional boundaries, offering a spectrum of tailored functionalities.

## 4 Conclusions

In conclusion, we successfully synthesized an ionic liquid-based silane and functionalized SiO_2_ nanoparticles used as a filler for a novel dental composite. Flexural strength was negatively affected by silane incorporation. However, all other physical and chemical tests showed stability or better results for the silanized materials. Furthermore, from 5 wt % of silane, an antibacterial effect was found, accentuating the higher silane concentration without affecting the cytotoxicity of the composite against human cells.

This study aimed to pioneer the integration of an ionic liquid into dental resin formulations replacing traditional organosilanes. Ionic liquids offer significant potential for their inherent antibacterial properties and versatile nature. Our objective was to create a material with chemical versatility, potentially targeting matrix metalloproteinases and bacterial or saliva enzymes and combating antibacterial resistance in the future. The authors suggest expanding the analyses to assess the effects of the synthesized ionic liquid on various bacterial species and biofilms, both immediately and over time.

## Supporting Information Available

Supporting Information 1 to 4 show the results of NMR data of ^13^C and ^29^Si techniques of the SiO_2_ nanoparticles silanized with the synthesized ionic liquid-based silane and the results of TEM and SEM analyses of these nanoparticles (PDF)
