# Supplementary material for: Ionic Liquid-Based Silane for SiO2 Nanoparticles: A Versatile Coupling Agent for Dental Resins
Source: ACS Appl Mater Interfaces. 2024 Jun 24;16(26):34057–68. doi: 10.1021/acsami.4c04580 (PMC11231966; doi:10.1021/acsami.4c04580)
Supplement: Supplementary file 1 — am4c04580_si_001.pdf [file am4c04580_si_001.pdf]

Supporting Information

Ionic liquid-based silane for SiO_2 nanoparticles: a versatile coupling agent for dental resins

Isadora Martini Garcia[a], Virgínia Serra de Souza[b], Abdulrahman A. Balhaddad[c], Lamia Mokeem[d], Mary Anne Sampaio de Melo[e], Jackson Damiani Scholten[f]*, Fabrício Mezzomo Collares[g]*

[a] Division of Cariology and Operative Dentistry, Department of Comprehensive Dentistry, University of Maryland School of Dentistry, Baltimore, MD 21201, USA. Dental Materials Laboratory, School of Dentistry, Federal University of Rio Grande do Sul. Porto Alegre, RS 90035-003, Brazil. E-mail: igarcia1@umaryland.edu. ORCID: https://orcid.org/0000-0002-7388-0200
[b] Laboratory of Molecular Catalysis, Institute of Chemistry, Federal University of Rio Grande do Sul. *Bento Gonçalves Avenue*, 9500, Agronomia, 91501-970, Porto Alegre, RS, Brazil. E-mail: vivi_qui@hotmail.com. ORCID: https://orcid.org/0000-0003-2750-5706
[c] Department of Restorative Dental Sciences, College of Dentistry, Imam Abdulrahman Bin Faisal University, P.O. Box 1982, Dammam 31441, Saudi Arabia. E-mail: abalhaddad@iau.edu.sa ORCID: https://orcid.org/0000-0001-6678-7940
[d] Dental Biomedical Sciences Ph.D. Program, University of Maryland School of Dentistry, Baltimore, MD 21201, USA. E-mail: lmokeem@umaryland.edu. ORCID: https://orcid.org/0000-0003-1071-1925
[e] Division of Cariology and Operative Dentistry, Department of Comprehensive Dentistry, University of Maryland School of Dentistry, Baltimore, MD 21201, USA. E-mail: Mmelo@umaryland.edu. ORCID: https://orcid.org/0000-0002-0007-2966
[f] Laboratory of Molecular Catalysis, Institute of Chemistry, Federal University of Rio Grande do Sul. Bento Gonçalves Avenue, 9500, Agronomia, 91501-970, Porto Alegre, RS, Brazil. E-mail: jackson.scholten@ufrgs.br. ORCID: https://orcid.org/0000-0002-7433-392X
[g] Department of Dental Materials, School of Dentistry, Federal University of Rio Grande do Sul. Ramiro Barcelos Street, 2492, Rio Branco, 90035-003, Porto Alegre, RS, Brazil. E-mail: fabricio.collares@ufrgs.br. ORCID: https://orcid.org/0000-0002-1382-0150

*** Corresponding authors:**
Fabrício Mezzomo Collares
fabricio.collares@ufrgs.br
Phone number: +55 51 33085198

Jackson Damiani Scholten
jackson.scholten@ufrgs.br
Phone number: +55 51 33089633

Table S1. NMR data of ^{13}C technique of the SiO_2 modified with different concentrations of the ionic liquid-based silane.

**Group**	**^{13}C NMR (ppm)**					
	**1, 2 [a]**	**3**	**4, 5 [a]**	**6**	**7**	**8**
SiO_2 without ionic liquid-based silane						
SiO_2 with 1% of ionic liquid-based silane						
SiO_2 with 2.5% of ionic liquid-based silane	128,8	135,4	124,6 120,4	51,1	21,7	8,2
SiO_2 with 5% of ionic liquid-based silane	129,2	136,0	123,2<br>119,4	50,9	23,8	8,6
SiO_2 with 7.5% of ionic liquid-based silane	128,6<br>108,4	135,0	121,2	50,0	23,2	8,9
SiO_2 with 10% of ionic liquid-based silane	129,3	135,3	124,0<br>121,9	50,5	24,3	9,2

[a] Superimposed signals.

Table S2. NMR data of ^{29}Si technique of the SiO_2 modified with different concentrations of the ionic liquid-based silane.

Group	**^{29}Si NMR (ppm)**				
	**1**	**2**	**3**	**4**	**5**
SiO_2 without ionic liquid-based silane	-108,9			-102,8	-99,0
SiO_2 with 1% of ionic liquid-based silane	-113,2	-52,2	-60,6		
SiO_2 with 2.5% of ionic liquid-based silane					
SiO_2 with 5% of ionic liquid-based silane	-107,4	-61,7	-66,7	-100,1	
SiO_2 with 7.5% of ionic liquid-based silane	-108,3	-58,6	-66,8	101,9	
SiO_2 with 10% of ionic liquid-based silane	-106,4	-58,4	-66,5		-99,1

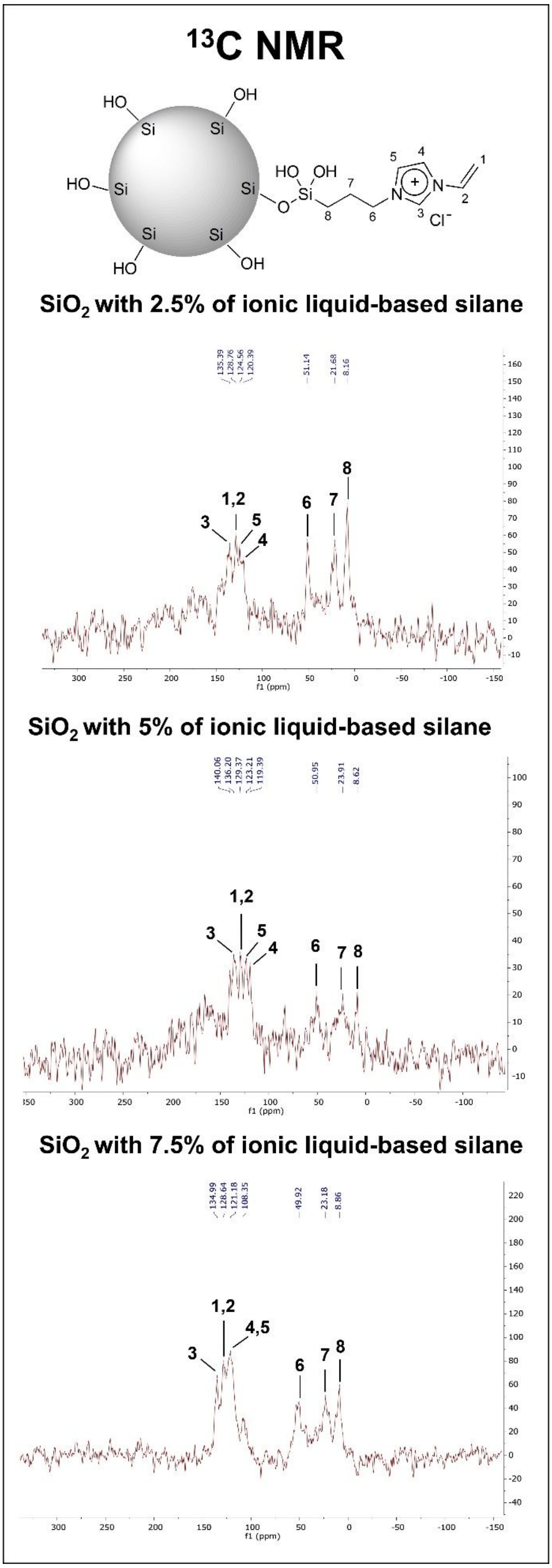

Figure S3. Spectra of ^{13}C NMR technique of the SiO_2 modified with different concentrations of the ionic liquid-based silane.

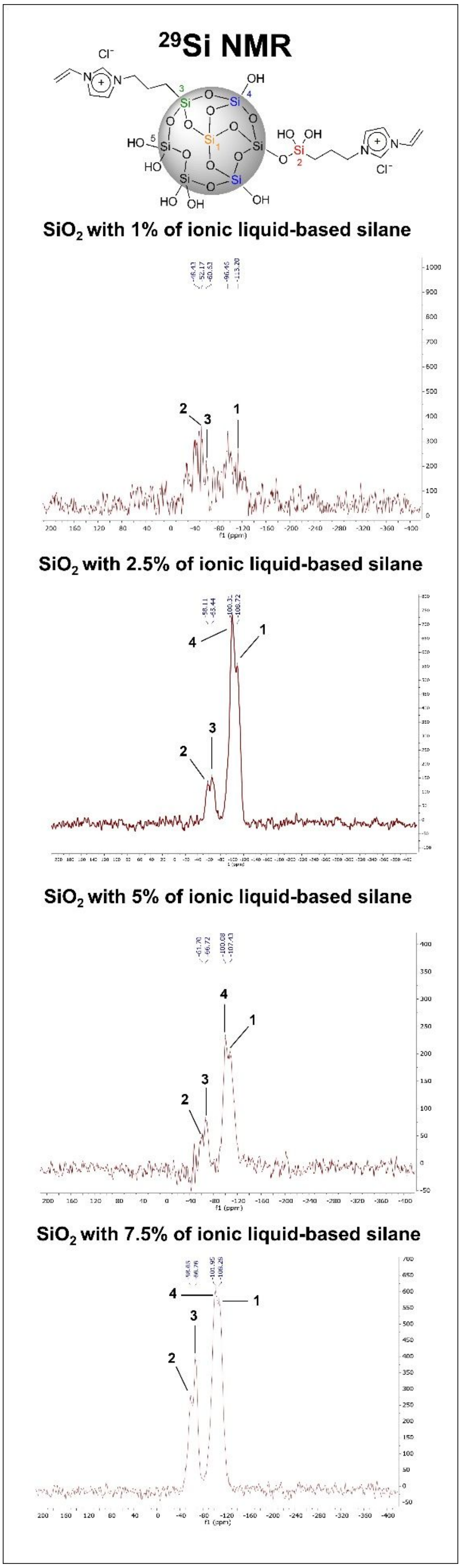

Figure S4. Spectra of ^{29}Si technique of the SiO_2 modified with different concentrations of the ionic liquid-based silane.

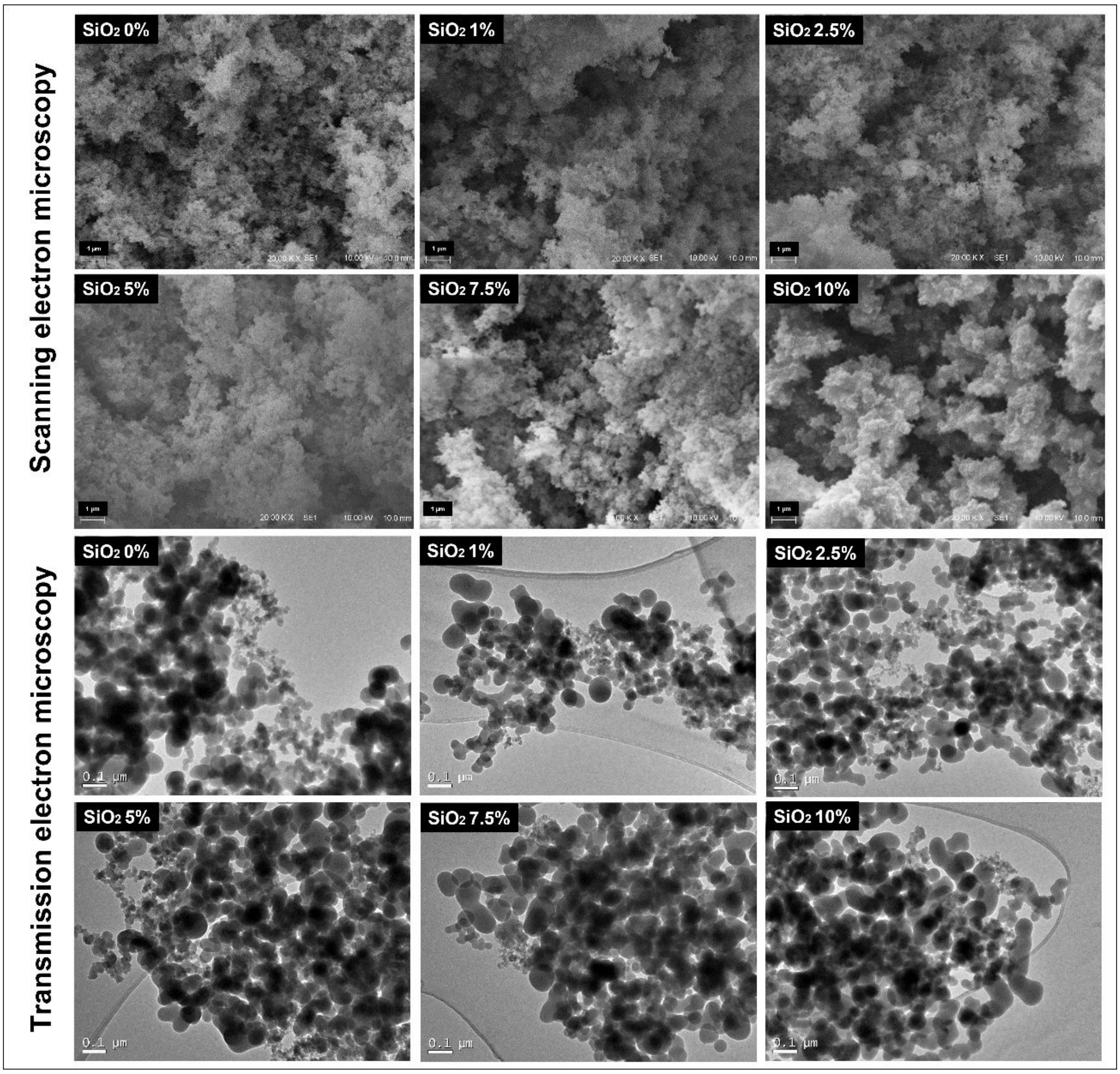

Figure S5. SEM and TEM images of the SiO_2 modified with different concentrations of the ionic liquid-based silane.
